# PRMT5-mediated histone H4 arginine-3 symmetrical dimethylation marks chromatin at G + C-rich regions of the mouse genome

**DOI:** 10.1093/nar/gkt884

**Published:** 2013-10-03

**Authors:** Michael Girardot, Ryutaro Hirasawa, Salim Kacem, Lauriane Fritsch, Julien Pontis, Satya K. Kota, Doria Filipponi, Eric Fabbrizio, Claude Sardet, Felix Lohmann, Shilpa Kadam, Slimane Ait-Si-Ali, Robert Feil

**Affiliations:** ^1^Institute of Molecular Genetics (IGMM), CNRS UMR 5535, University of Montpellier, 1919 route de Mende, 34293 Montpellier, ^2^Laboratoire Epigénétique et Destin Cellulaire, UMR7216, CNRS, Université Paris Diderot, 35 rue Hélène Brion, 75013 Paris, France and ^3^Developmental and Molecular Pathways, Novartis Institutes for BioMedical Research, 250 Massachusetts Avenue, Cambridge, MA 02139, USA

## Abstract

Symmetrical dimethylation on arginine-3 of histone H4 (H4R3me2s) has been reported to occur at several repressed genes, but its specific regulation and genomic distribution remained unclear. Here, we show that the type-II protein arginine methyltransferase PRMT5 controls H4R3me2s in mouse embryonic fibroblasts (MEFs). In these differentiated cells, we find that the genome-wide pattern of H4R3me2s is highly similar to that in embryonic stem cells. In both the cell types, H4R3me2s peaks are detected predominantly at G + C-rich regions. Promoters are consistently marked by H4R3me2s, independently of transcriptional activity. Remarkably, H4R3me2s is mono-allelic at imprinting control regions (ICRs), at which it marks the same parental allele as H3K9me3, H4K20me3 and DNA methylation. These repressive chromatin modifications are regulated independently, however, since PRMT5-depletion in MEFs resulted in loss of H4R3me2s, without affecting H3K9me3, H4K20me3 or DNA methylation. Conversely, depletion of ESET (KMT1E) or SUV420H1/H2 (KMT5B/C) affected H3K9me3 and H4K20me3, respectively, without altering H4R3me2s at ICRs. Combined, our data indicate that PRMT5-mediated H4R3me2s uniquely marks the mammalian genome, mostly at G + C-rich regions, and independently from transcriptional activity or chromatin repression. Furthermore, comparative bioinformatics analyses suggest a putative role of PRMT5-mediated H4R3me2s in chromatin configuration in the nucleus.

## INTRODUCTION

DNA methylation is involved in diverse epigenetic phenomena in mammalian development, including X-chromosome inactivation and genomic imprinting (1–3). Similarly, histone lysine methylation plays diverse roles in the establishment and maintenance of functional chromatin states during development (4). Different arginine residues on histones H2A, H3 and H4 can also be mono- or dimethylated (5). Previously, we and others have shown the association of H4 arginine-3 symmetrical dimethylation (H4R3me2s) with specific repressed loci in mammals (5–11). These findings evoked possible roles of H4R3me2s in gene regulation, but also emphasized the need to further explore its regulation and genome-wide distribution.

Imprinted gene expression in mammals is mediated by ‘imprinting control regions’ (ICRs), CpG-rich regulatory sequences that are marked by DNA methylation on one of the two parental alleles only (3,12). Besides differential histone lysine methylation and acetylation, we and others detected H4R3me2s at ICRs and at intracisternal A particles (IAPs) in mouse embryos (7,13,14). This observation suggested that H4R3me2s could be linked to gene repression. Consistent with this model, earlier reports had shown enrichment of H4R3me2s at different repressed genes, including the rDNA, hemoglobin beta (HBB) and Cyclin E1 genes (6,8,11). However, genome-wide analyses of the distribution of this covalent histone mark were missing to test this idea. It also remained unclear which protein arginine methyltransferase(s) (PRMT) could control the H4R3me2s mark in mammalian somatic cells. In the current study, we explored these key questions in mouse embryonic fibroblasts (MEFs) and embryonic stem (ES) cells. Using an shRNA-mediated gene knock-down approach, we demonstrate that the type-II protein arginine methyltransferase PRMT5 controls the bulk of H4R3me2s in MEFs. Chromatin immunoprecipitation (ChIP) followed by next-generation DNA sequencing (ChIP-seq) was used to delineate the genome-wide distribution of H4R3me2s in both ES and MEF cells. The latter analysis revealed that, rather unexpectedly, H4R3me2s is enriched preferentially at DNA sequences with a high G + C content, including ICRs. Using imprinted gene loci as a model system, we carefully explored the relationship of H4R3me2s with other histone modifications in somatic cells. Finally, using comparative bioinformatics analyses we found interesting correlations with factors involved in higher order chromatin contacts.

## MATERIALS AND METHODS

### Primary cells, immuno-cytochemistry and RT-polymerase chain reaction analysis

Primary MEFs used for *Prmt5* and *Eset* knockdown were established from E13.5 embryos, obtained by crossing C57BL6/J (*Mus musculus domesticus*) females with JF1 (*Mus musculus molossinus*) males (15). The ES cells (line BJ1) used for immunoblotting and immunocytochemistry were newly established in serum-free 2i medium from blastocysts obtained by mating a C57BL6/J, *Oct4*-GFP transgenic female (16) with a JF1 male. Their pluripotency is described elsewhere (Kota *et al.*, in preparation). *Eset* knockout ES cells and *Suv4-20h1/h2* double-knockout MEFs were derived by Lohmann *et al.* (17) and Schotta *et al.*(18), respectively. Total RNA was isolated using TRIzol reagent (Invitrogen) and cDNA was synthesized using SuperScript-2 reverse transcriptase (Invitrogen) and random oligonucleotides. Primers used for polymerase chain reaction (PCR) are described in Supplementary Table S2. For immunocytochemistry, cells were seeded on gelatin-coated cover slips, fixed for 10 min in 4% paraformaldehyde in phosphate buffered saline (PBS) at room temperature, and washed with PBS. After 30-min incubation with pre-treatment buffer (1% horse serum, 2% Triton-X100 in PBS), cells were incubated for 3 h with specific antisera at RT. After extensive washes, they were incubated for 30 min at RT with an appropriate Alexa488-conjugated second antibody. Stained cells were mounted in Vectorshield mounting medium (Vector Laboratory) containing DAPI for counterstaining of DNA, and were microscopically analysed.

### Western blotting

Whole cell lysates, or protein extracts, were resolved on sodium dodecyl sulphate–polyacrylamide gel electrophoresis (SDS–PAGE) gels or on pre-cast NuPage 4–12% acrylamide gradient SDS–PAGE gels (Invitrogen), and transferred onto nitrocellulose membrane. Membranes were blocked and incubated overnight at 4°C with primary antibodies. After incubation with appropriate secondary antibodies coupled to horseradish peroxidase, images were developed using the West Dura kit and ChemiSmart 5000 system (Vilber Laurmat). For producing PRMT5- and PRMT7 over-expressing 293T cells, full-length cDNAs cloned into the vector pCMV-SPORT6 (Open Bioscience) were used.

### ChIP and PCR analysis of precipitated chromatin

ChIP on native chromatin was performed following a previously described protocol (19) with the following modifications. The purified nuclei were incubated for 15 min at 37°C with 60 units of Micrococcal nuclease (S7 Micrococcal nuclease, from *Staphylococcus aureus*, Roche) to produce mono/di-nucleosomes as verified by agarose gel electrophoresis. Nucleosomes were precipitated with the H4R3me2s antiserum-2 (Supplementary Table S1) and DNA was purified from precipitated chromatin fractions with the ‘ChIP DNA purification kit’ (Zymo Research). For ChIP on cross-linked chromatin, cells were fixed with 1% formaldehyde for 10 min, and quenched with 125 mM of glycine. Subsequently, cells were washed in PBS, collected in 15-ml tubes and centrifuged to remove the supernatant. Cells were re-suspended in 100 µl of SDS lysis buffer (100 mM NaCl, 1% SDS, 5 mM ethylenediaminetetraacetic acid (EDTA), 50 mM Tris–Cl and ‘Complete protease inhibitor cocktail’ from Roche). After 10 min on ice with occasional vortexing, cells were diluted in 200 µl of ice-cold ChIP dilution buffer (0.01% SDS, 1.1% Triton X-100, 1.2 mM EDTA, 16.7 mM Tris–Cl, 167 mM NaCl and ‘Complete protease inhibitor cocktail’ from Roche), and sonicated twice at high power for 30 cycles of 30 s ON/ 30 s OFF in a BioRuptor twin apparatus (Diagenode). The average DNA fragments’ length from ES and MEF cells was ∼200 bp, as estimated by agarose gel electrophoresis. Q-PCR on purified DNA was performed using the LightCycler® 480 SYBR Green I Master Mix (Roche) and LigthCycler 480 apparatus (Roche). Antisera and oligonucleotides used are listed in Supplementary Tables S1 and S2.

### High-throughput sequencing and bioinformatics

Input and ChIPed DNAs were sequenced on an Illumina Hi-seq 2000 instrument (Fasteris, Switzerland) using the TruSeq™ SBS v3 kit. The sequences produced from the input chromatin and H4R3me2s ChIPed DNA from ES and MEF cells are detailed in the Supplementary Table S3. ChIP-seq raw data (fastq) were aligned on the *M. musculus* reference genome (mm9) using the Bowie 0.12.7 software. Smoothed tag density compared to input tag density were computed with the Sequence processing pipeline (20) and visualized with the integrative Genomics Viewer (21). Median tag density around the defined peak positions were calculated using an in-house designed R-script. Briefly, peaks’ summits coordinates were grouped according to the level of enrichment for the mark examined and median tag densities were calculated every 100 bp across 5 kb windows around the peaks’ summits. Heatmaps of H4R3me2s were clustered and represented using the seqMINER 1.3.3 program (22). H3K4me3 and H3K4me1 peaks generated by Dr Bing Ren’s laboratory were downloaded from the UCSC genome browser (http://genome.ucsc.edu/cgi-bin/hgFileUi?db=mm9&g=wgEncodeLicrHistone). Peak predictions for H4R3me2s and PRMT5 in the two cell types were performed with the Model-based Analysis for ChIP-seq (MACS) 1.4.2 algorithm with the default parameters (*P* < 1e^-5^) (23). 100-bp windows with the same H4R3me2s enrichment in MEF and ES cells were grouped together and represented as a density array using the hexbin package. The genome-wide G + C frequency was calculated using the LetterFrequencyInSlidingView function from the Biostrings package (http://www.bioconductor.org/packages/2.11/bioc/html/Biostrings.html) in R 2.13.0 (http://www.R-project.org/). The MeDIP and hMeDIP-seq in ES cells were from Ficz *et al.* (24) (http://www.ebi.ac.uk/ena/data/view/PRJEB2462). Published ChIP-seq profiles for H3K4me3, H3K27me3 and RNA-seq from ES cells cultivated in 2i serum-free medium (25) were retrieved from the GEO database (GSE23943). Genomic coordinates for repeat elements were retrieved from the RepeatMasker database (http://www.repeatmasker.org/).

### shRNA preparation and transfection

*Prmt5* shRNAs were designed against unique sequences such as to ensure that only the *Prmt5* mRNA was targeted: 5′-GAG GGA GTT CAT TCA GGA A-3′ (shPrmt5-1) and 5′-GGA TGT GGT GGC ATA ACT T-3′ (shPrmt5-2). The sh-Ctrl targets a non-genomic firefly luciferase sequence (26). Similarly, sh-*Eset-1* targets a unique sequence in *Eset* mRNA, 5′-CAGTTCTCAAGATCTACAT-3′. shRNA sequences were cloned into the retroviral vector RNAi Ready pSiren (BD Biosciences) according to the manufacturer’s instructions. Retroviral vector and packaging vectors (gag-pol and VSV-G) were co-transfected into 293T cells using the calcium phosphate method. After 3 days incubation, supernatants were collected, filtrated and used for infection. Primary MEFs at passages 2-3 were incubated for 48 h with virus particles and polybrene (Millipore). After medium change, cells were selected in 20 μg/ml puromycin for 48 h. Selected cells were incubated with fresh medium for 3 days and then used for analyses.

### Data access

The ChIP-seq data from this publication have been submitted to the GEO database http://www.ncbi.nlm.nih.gov/geo and assigned the identifier GSE37604.

## RESULTS

### PRMT5 controls arginine-3 symmetrical dimethylation on H4 in embryonic cells

**Figure 1.**
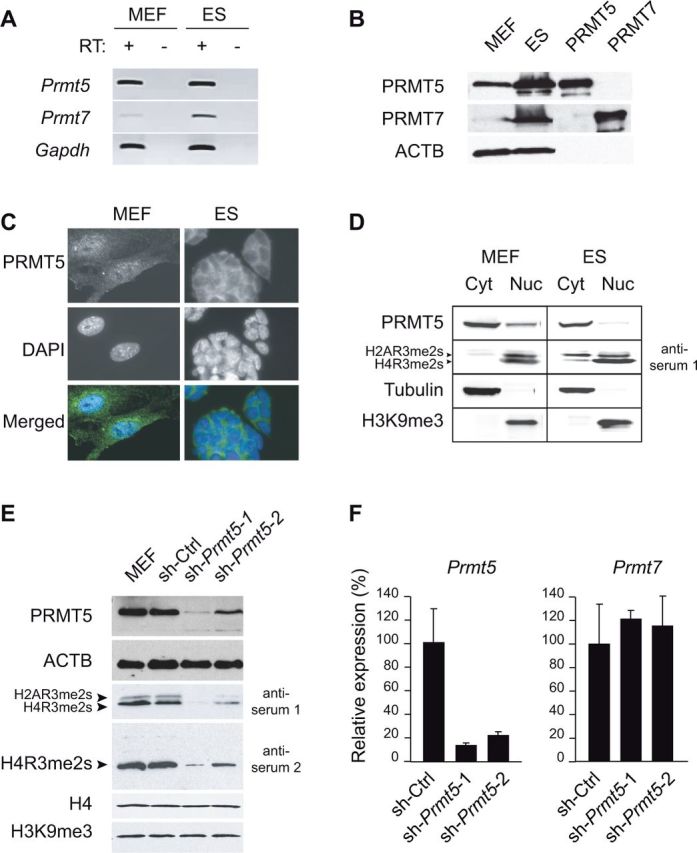
PRMT5 expression, localization and histone methylation activity in embryonic cells. (**A**) RT-PCR analysis of *Prmt5* and *Prmt7* expression in primary MEFs and ES cells. RT+ and RT– indicate presence and absence of reverse transcriptase (RT), respectively. (**B**) Western blot analysis of total protein extracts. Lanes ‘PRMT5’ and ‘PRMT7’ were loaded with diluted protein samples of 293T cells in which the PRMT5 and PRMT7 proteins were over-expressed. β-Actin (ACTB) is used as a loading control. (**C**) Immunofluorescence staining of PRMT5 in MEFs and ES cells (upper panels). Nuclei were counter-stained with DAPI (middle panels). (**D**) Western blot analysis of cytoplasmic (Cyt) and nuclear (Nuc) protein fractions of MEFs and ES cells. Tubulin and H3K9me3 constitute cytoplasmic and nuclear controls, respectively. (**E**) Strongly reduced PRMT5 protein expression in MEFs stably infected with lentiviral shRNA constructs (sh-Prmt5-1 and sh-Prmt5-2) directed against *Prmt5*. As a negative control, MEFs stably expressing a scrambled shRNA (Sh-*Ctrl*) were analysed. PRMT5, H4R3me2s (anti-serum 2) and H2A/H4R3me2s levels (anti-serum 1) were assessed by western blotting of total protein extracts; ACTB provides a loading control. (**F**) Strongly reduced *Prmt5* gene expression in sh-Prmt5-1 and sh-Prmt5-2 cells. cDNA was made from total RNAs using random oligonucleotides. Expression levels were determined relative to *Gapdh* by real-time PCR amplification, and were put arbitrarily at 100% in the control cells (Sh-*Ctrl*).

First, we sought to determine which enzyme(s) control(s) H4R3me2s (and H2AR3me2s whose first 8 residues are 88% identical) in primary MEFs. We explored the type-II PRMTs candidate for this type of symmetrical dimethylation: PRMT5 and PRMT7 (5). Since PRMT5 had been reported to control H2AR3me2s in ES cells (27), we also studied ES cells that were newly derived in 2i-medium (28), and which were of the same genotype as the primary MEFs. *Prmt5* mRNA expression was readily detected in both MEFs and ES cells and in all somatic tissues analysed (Figure 1A, Supplementary Figure S1A), suggestive of ubiquitous expression. *Prmt7*, in contrast, was poorly expressed in MEFs, but showed relatively high expression in ES cells and in male and female gonads (Supplementary Figure S1A). Similarly, western analyses showed high expression of PRMT5 protein in MEFs and ES cells, whereas PRMT7 was barely detectable in MEFs, but was highly expressed in ES cells (Figure 1B). These data pinpoint PRMT5 as the main type-II PRMT in MEFs.

PRMT5 had been reported to be mostly cytoplasmic in ES cells (27). We confirm this finding by immunostaining of fixed cells and western blot analyses of cytoplasmic and nuclear protein fractions (Figure 1C and D). In MEFs, in contrast, PRMT5 is readily detectable in the nucleus (Figure 1C and D). In both cell types, H2AR3me2 and H4R3me2s are detected in the nucleus. In ES cells, however, part of the H2AR3me2s signal is present in the cytoplasm as well, a finding which agrees with the proposition that in undifferentiated cells PRMT5 may pre-modify this core histone already in the cytoplasm (27). In MEFs, in contrast, H2AR3me2 and H4R3me2s are exclusively nuclear, suggesting that in differentiated cells, PRMT5 targets histones H2A and H4 predominantly in the nucleus. Combined, our data pinpoint PRMT5 as the candidate type-II enzyme that modifies nuclear histones in MEFs.

Next, to explore the importance of PRMT5 in histone arginine methylation, we knocked down its expression in primary cells in culture. In an earlier study, it had been found that complete genetic ablation of *Prmt5* in the mouse was not compatible with cell survival during early development (27). This deleterious knockout phenotype could be related to multiple known roles of PRMT5, unrelated to chromatin, including the biogenesis and action of SMN–Sm protein complex, RNA splicing and signalling (29,30). To reduce, rather than completely abrogate expression, we designed retroviral shRNA constructs directed against *Prmt5*. Two lentiviral constructs, sh-*Prmt5*-1 and sh-*Prmt5*-2, were stably introduced into primary MEFs, with >80% of cells surviving 48 h after viral infection and selection for shRNA expression (data not shown). A construct with a scrambled DNA sequence, Sh-Ctrl, was used as a negative control in these experiments. Both sh-*Prmt5*-1 and sh-*Prmt5*-2 led to reduced PRMT5 mRNA and protein expression after 72 h of growth in selective medium (to ablate non-infected cells not expressing the shRNA) (Figure 1E and F). The expression of the other type-II PRMT, *Prmt7*, was unaffected (Figure 1F). In agreement with the essential roles of PRMT5, we observed reduced cellular proliferation in PRMT5-depleted cells (Supplementary Figure S1B). Significantly, the reduction in PRMT5 protein levels led to a global reduction in H4R3me2s and H2AR3me2s (∼85% reduction for Sh-*Prmt5*-1), as detected by western blotting with a specific antiserum that recognizes both histones (Figure 1E, ‘antiserum 1’). This finding was confirmed with an independent second antiserum (Figure 1E, ‘antiserum 2’), which only recognized H4R3me2s, again showing ∼85% reduction for this histone modification. Importantly, in the PRMT5-depleted cells, overall histone H4 levels and histone H3 lysine-9 trimethylation (H3K9me3) appeared unaffected (Figure 1E). We conclude from these data that PRMT5 controls H4R3me2s in MEF cells.

### H4 arginine-3 symmetrical dimethylation is enriched at G + C-rich sequences of the genome

We next performed ChIP-seq assays to identify the global genomic distribution of H4R3me2s. Before performing ChIP-sequencing, however, we further assessed the quality of ‘antiserum 2’ directed against H4R3me2s (Supplementary Table S1). This antiserum did not recognize H2AR3me2s (Figure 1E), nor asymmetrical dimethylation or monomethylation of H4R3 (31). The immunofluorescence staining on fixed cells with this antiserum was mostly nuclear in MEF cells (Supplementary Figure S2A). Using a peptide array of histone modifications, we confirmed that antiserum 2 was highly specific for the H4_(1-19)_R3me2s modification (Supplementary Figure S2B) and that its binding was not altered by the presence of neighbouring modifications (namely, S1p, K5ac, K8ac and K12ac of H4) (Supplementary Figure S2C). Treatment of cells with the HDAC inhibitor Trichostatin-A (TSA) did not change the antibody binding of H4R3me2s in western blotting analyses (Supplementary Figure S2D), confirming that gain of acetylation at nearby residues (H4K5, H4K8) in living cells did not significantly impair recognition of H4R3me2s by the ‘antiserum 2’. Importantly, the *Prmt5* knock-down in MEF cells led to an almost complete loss of H4R3me2s detection with this antiserum (Figure 1E), providing another indication of its specificity. Finally, six promoters were chosen randomly (Supplementary Figure S2E) to perform ChIP assay in the absence or in the presence of an H4R3me2s-blocking peptide (Supplementary Figure S2F). We observed strongly reduced immunoprecipitation of H4R3me2s in the presence of an H4R3me2s-blocking peptide (Supplementary Figure S2F), which further validates the choice of ‘antiserum 2’ for subsequent ChIP-seq studies.

**Figure 2.**
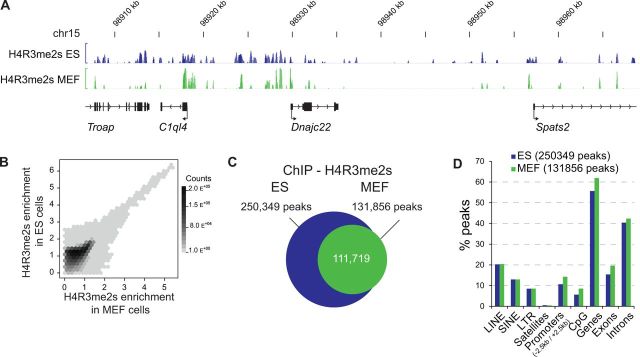
Genome-wide analyses of H4R3me2s in mouse ES cells and MEFs. (**A**) Representative ChIP-Seq data along a 65-kb window on mouse chromosome 15; normalized tag densities for H4R3me2s in ES and MEF are represented. Gene positions are indicated below. (**B**) Genome-wide correlation between H4R3me2s-enriched regions in ES (*y*-axis) and MEF (*x*-axis) cells. The scales of the *x*- and *y*-axis are transformed by the following formula: Log10(1 + x). Genomic regions (100-bp windows) with defined degrees of ES and MEF H4R3me2s enrichments are plotted as hexagonal tiles. The number of genomic regions in each hexagon is depicted by a grey colour scale of increasing darkness, according to the count scale reported on the right. (**C**) Venn diagram representing the number of MACS-predicted peaks (*P* < 1e^-5^) of H4R3me2s in ES and MEF cells. The intersection of the areas representing ES and MEF cells indicates peaks common to both cell types. (**D**) Comparisons of the localizations of ES and MEF’s H4R3me2s enrichments peaks. In both cell types, most peaks are located at genes.

Whereas H3K9me3 showed a punctuated staining pattern, mostly confined to heterochromatic foci, the staining of H4R3me2s on fixed MEF cells was homogenous in the nucleus (Supplementary Figure S2A), suggesting that this histone arginine methylation could be broadly present along the genome. To explore the genomic distribution pattern of H4R3me2s, we performed ChIP-seq on native chromatin from mouse embryonic cells, using the antiserum 2. Indeed, we found that H4R3me2s displayed a broad pattern of precipitation, with many peaks along the genome, similarly in ES and MEF cells. As an example of this unique epigenomic pattern, a representative 65-kb genomic window is shown in Figure 2A. Significantly, the majority of the H4R3me2s-marked regions were common between MEFs and ES cells (Figure 2B) (Pearson’s *r* = 0.98). Next, we defined the peaks of H4R3me2s enrichment over input chromatin that had a high statistical probability (*P*-value cut-off for peak detection by MACS: *P* < 1e^-5^). No fewer than 250 349 ‘high-probability peaks’ were thus identified in ES cells and 131 856 in MEFs cells. Interestingly, the vast majority of the high probability peaks in MEFs were also present in ES cells (Figure 2C) and their genomic localization is similar in the two cell types (Figure 2D), with a predominant accumulation at genes. Noteworthy, we did not detect enrichment in sequence reads for satellite sequences in H4R3me2s ChIP compared with input chromatin (Supplementary Table S3). However, we detected significant H4R3me2s enrichments in repetitive elements such as LINEs (long interspersed nuclear elements), SINEs (short interspersed nuclear elements) and LTRs (long terminal repeats, including IAPs).

**Figure 3.**
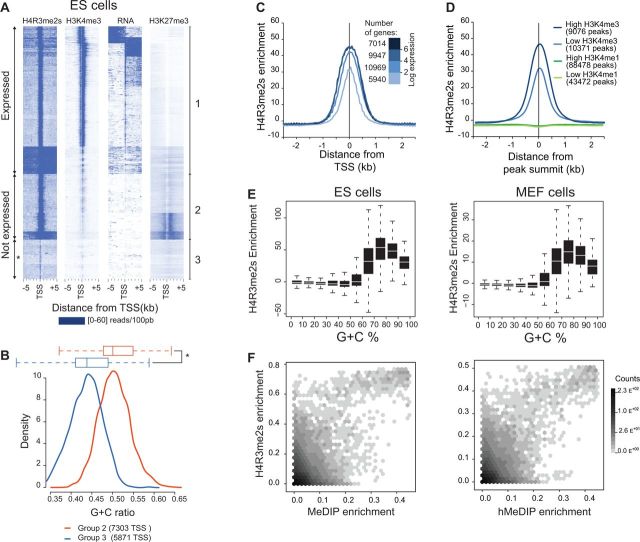
H4R3me2s accumulates at promoters and other G + C-rich regions, generally irrespective of transcriptional activity. (**A**) The unsupervised clustering of transcriptional start sites (TSSs) is represented as a heatmap of tag densities (0–60) per 100-bp bins, from 5 kb upstream to 5 kb downstream of mouse TSS (*n* = 33 876) for the indicated ChIP-seq/RNA-seq experiments in ES cells. ChIP-seq profiles for H3K4me3, H3K27me3 and RNA-seq from ES are from Marks *et al.* (25). The non-expressed class of TSS marked by an asterisk contains genes expressed in differentiated cells such as genes belonging to the immune response, inflammatory response or locomotory behaviour Gene Ontology terms. (**B**) The two class of TSS (groups 2 and 3) indicated in (A) were analysed for their G + C ratio. The two classes of promoters are represented as density plot and box-and-whisker plots in red and blue, for the groups 2 and 3, respectively. (*) Indicates Wilcoxon rank sum test with a *P* < 2.2e^-16^. (**C**) H4R3me2s enrichment around TSSs grouped into four different classes according to the genes’ log expression values. (**D**) Mean H4R3me2s enrichment levels in ES cells are represented as a function of their distance from the peak summits of H3K4me3 or H3K4me1 peaks. H4R3me2s is enriched around H3K4me3 peaks, but not around H3K4me1 peaks. However, H4R3me2s enrichment occurs both at H3K4me3-enriched regions with high H3K4me3 levels (high H3K4me3) and those with lower H3K4me3 enrichment (low H3K4me2). (**E**) H4R3me2s enrichment according to the G + C content of 100-bp genomic windows in ES and MEF cells showing the absence of H4R3me2s in regions with less than 50% G + C ratio. (**F**) Hexagonal binning of H4R3me2s-enriched regions according to their DNA methylation level assessed by MeDIP, or hydroxymethylation level assessed by hydroxyMeDIP (hMeDIP), in ES cells (24). The scales of the *x*- and *y*-axis are transformed by the following formula: asinh^-1^(x). Darker shades of grey indicate higher densities of bins as reported on the count scale.

We next asked whether H4R3me2s enrichment correlated with either gene expression or gene repression. Remarkably, 29 539 (87.2% of total) and 22 741 (67% of total) promoters in ES and MEF cells, respectively, were enriched in H4R3me2s. This indicates that the majority of all annotated promoters are marked by H4R3me2s (Figure 3A). Importantly, promoters that are enriched for H3K4me3 and that are actively transcribed (RNA-seq) (Figure 3A, group 1) are similarly enriched for H4R3me2s as transcriptionally inactive promoters (group 2) that show low H3K4me3 and high H4K27me3 enrichments. Promoters that were statistically enriched in H4R3me2s in ES cells, but not in MEFs, included genes expressed in undifferentiated ES cells (and the early embryo), but not in differentiated cells, such as *Dnmt3L, Dnmt3A, PiwiL1* and *Tdrd1*. Also the *Prmt7* gene was highly marked by H4R3me2s in ES cells, but much less so in MEFs. The silenced gene promoters that showed little H4R3me2s enrichment (marked by an asterisk in Figure 3A) in ES cells included immune response genes such as the *CD4 antigen* (*Cd4*) and *Interferon beta 1* (*Ifnb1*) genes.

Interestingly, we found that the H4R3me2s-negative inactive promoter sequences (group 3; *n* = 5871) were significantly less G + C-rich (mean G + C ratio = 0.43) than the H4R3me2s-positive inactive promoters (group 2; *n* = 7303) (mean G + C ratio = 0.50) (Wilcoxon rank sum test with a *P* < 2.2e^-16^) (Figure 3B). This finding provided a first indication that sequence context could be determinant in guiding H4R3me2s levels (see below). Importantly, we did not observe differences in H4R3me2s enrichment between four different classes of expressed genes that were grouped according to their expression levels in ES cells (Figure 3C). Thus, gene promoters were generally marked independently of their levels of expression. Concordantly, H4R3me2s co-localized with H3K4me3-enriched regions in the genome, which are mostly confined to active promoters (Encode/LICR dataset with the UCSC reference number wgEncodeEM001455) (Figure 3D). In contrast, no co-localization was observed with H3K4me1-enriched regions (Figure 3D), which include enhancers, nor with H3K9me3-marked regions (data not shown), which include heterochromatin and stably repressed genes. Examples of the lack of correlation between transcriptional activity and H4R3me2s enrichment are provided by the *Oct4* (*Pou5f1*) and *Nanog* genes, which are expressed in undifferentiated ES cells but not in MEFs (Supplementary Figure S2G-H). In both the cell types, however, the promoters of these pluripotency genes were strongly marked by H4R3me2s. Similar examples are provided by the *Col1a1* and *Actb* genes (Supplementary Figure S2I-J).

Most promoter regions have a high G + C content and many correspond to CpG islands (32). We therefore asked whether H4R3me2s could be enriched at G + C-rich sequence elements in general. Specifically, we evaluated H4R3me2s levels of 100-bp windows along the mouse genome ranked according to their G + C content. Remarkably, enrichment of H4R3me2s was confined to regions with a G + C content of more than 50%. Regions of low G + C content did not show any H4R3me2s. This strong link between G + C content and H4R3me2s enrichment was observed both in MEFs and ES cells (Figure 3E).

G + C-rich sequences comprise CpG dinucleotides at which the cytosine can be methylated or not. Therefore, we explored whether levels of 5m-Cytosine methylation or 5-hydroxymethylation in ES cells correlated with the observed enrichment levels of H4R3me2s. We found a weak correlation between the level of CpG methylation and H4R3me2s (Pearson’s *r* = 0.3). A stronger correlation was found with 5-hydroxymethylation of Cytosines (Pearson’s *r* = 0.33), which is associated with increased transcriptional activity (24) (Figure 3F). However, strong H4R3me2 enrichment was detected both at DNA-methylated and at unmethylated G + C-rich sequences. Accordingly, we observed H4R3me2s enrichment in ES and MEFs at the *Oct4* and *Nanog* promoters, which are unmethylated in ES cells and methylated in the E14.5 MEFs, indicating that H4R3me2s is independent of 5m-Cytosine at these genes (Supplementary Figure S2G-H). Similar findings were reported in resting human B lymphocytes at rDNA genes, at which H4R3me2s is enriched both at DNA-methylated and unmethylated copies (6). Combined, these data indicate that H4R3me2s is a hallmark of G + C-rich sequence elements, but is generally independent of transcriptional levels or DNA methylation.

### ICRs are marked by parental allele-specific H4R3me2s

**Figure 4.**
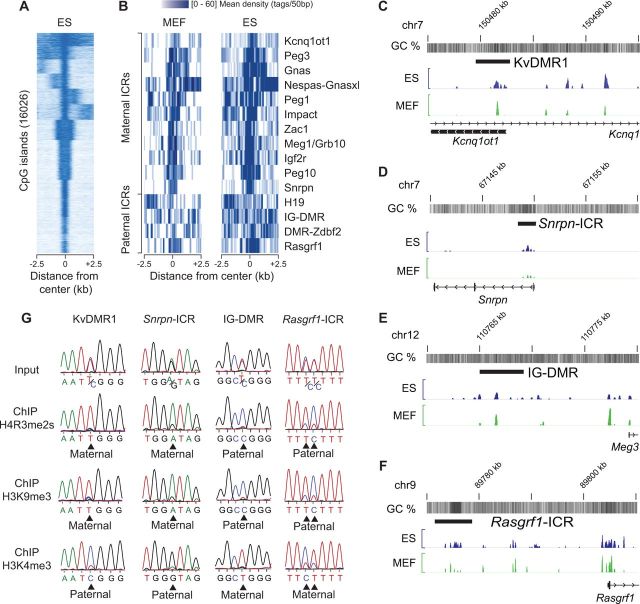
Allelic H4R3me2s enrichment at ICRs. (**A**) The unsupervised clustering of non-oriented CpG islands (*n* = 16 026) is represented as a heatmap of H4R3me2s enrichment levels in mouse ES cells in a window of 5 kb around the centre of each CGIs (the minimum to maximum lengths of CGIs are 201–5129 bp with an average length of 655 bp). (**B**) Heatmap representation of H4R3me2s enrichments in MEF and ES cells at ICRs, which are DNA-methylated on the maternal (‘maternal ICRs’) or the paternal (‘paternal ICRs’) chromosome. (**C–F**) ChIP-seq profiles for H4R3me2s, in ES cells and MEFs at the KvDMR1 (C), *Snrpn* (D), IG-DMR (E) and *Rasgrf1* (F) ICRs. (G) Sequence profiles around single nucleotide polymorphisms (SNPs) at the indicated ICRs. The parental alleles are equally present in the input chromatin used for ChIP (Input). H4R3me2s, H3K9me3 and H3K4me3 are preferentially enriched at one parental allele indicated by an arrow and the identified parental allele at ICRs. H3K4me3 is present on non-methylated alleles, while H4R3me2s and H3K9me3 are present on DNA-methylated alleles of ICRs.

CpG islands are particularly rich in G + C nucleotides (G + C content >50%, length >200 bp and observed/expected CpG >0.6) and they are all marked by H4R3me2s (Figure 4A). One class of CpG islands corresponds to the ICRs: the regulatory elements which mediate imprinted mono-allelic gene expression in mammals (33). Imprinted CpG islands are exceptional in that they are DNA-methylated on one of the two parental alleles only; and this differential DNA methylation is maintained throughout development. We find that both in ES cells and MEFs, ICRs are marked by peaks of H4R3me2s (Figure 4B–F). Previously, we reported for whole embryos, that H4R3me2s was present preferentially on the DNA-methylated alleles of ICRs (7). To determine whether this is also the case in primary MEFs, ChIP was performed on MEFs that were generated from embryos that were hybrid between *M. m. domesticus* strain C57BL6 and *M. m. molussinus* strain JF1. For this allele-specific ChIP assay, we generated larger native chromatin fragments of 2–7 nucleosomes in length. Single nucleotide polymorphisms (SNPs) allowed us to directly distinguish the parental chromosomes in the precipitated chromatin fractions (see Supplementary Table S2). At four different ICRs analysed, PCR amplification of ChIPed DNA followed by DNA sequencing clearly showed that H4R3me2s was precipitated on the DNA-methylated allele predominantly. The opposite, unmethylated allele was largely devoid of H4R3me2s (Figure 4G). These results were confirmed by PCR amplification followed by electrophoretic detection of single-strand conformation polymorphisms (SSCPs) (data not shown). We conclude from these data that H4R3me2s marks the DNA-methylated alleles of ICRs in MEFs. Since chromatin on the unmethylated alleles of ICRs is enriched in histone H4 acetylation, this finding also confirms that the antiserum used does not cross-react with acetylated H4.

### PRMT5 controls the parental allele-specific H4R3me2s at ICRs

**Figure 5.**
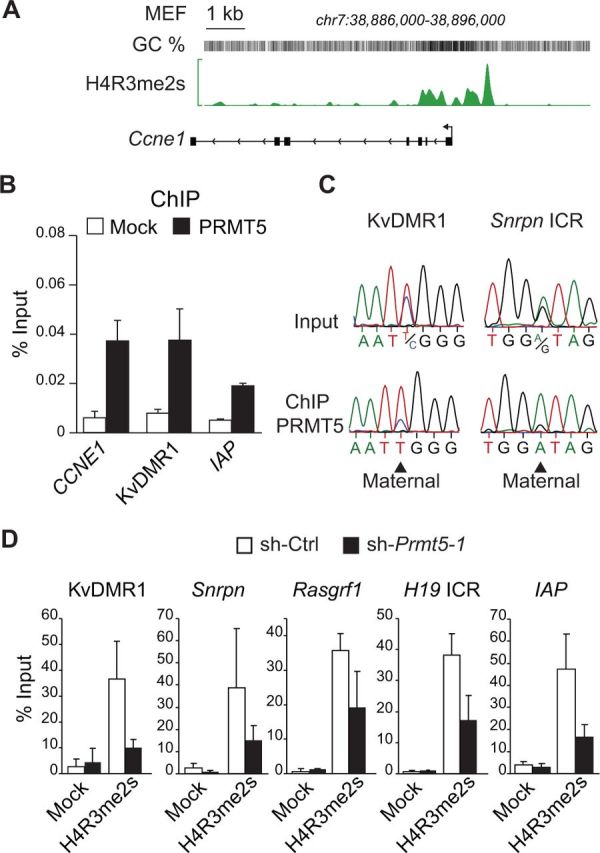
PRMT5 recruitment correlates with H4R3me2s enrichment at specific target loci. (**A**) ChIP of PRMT5 on cross-linked chromatin from MEFs. The *CyclinE1* (*Ccne1*) promoter is bound by PRMT5 and enriched for H4R3me2s in MEF cells, in agreement with previous studies (10). Shown are real-time PCR quantifications of PRMT5 association at *Cyclin E1* (*Ccne1*), the KvDMR1 ICR and IAP elements. (**B**) Direct DNA sequencing of ChIP samples shows PRMT5 binding to the H4R3me2s-marked (and DNA-methylated) allele of the KvDMR1 and *Snrpn* ICRs. (**C**) Real-time PCR amplification shows reduced H4R3me2s at ICRs and at IAP elements in PRMT5-depleted MEFs (sh-*Prmt5*-1 cells). ChIP was performed on native chromatin. In a mock ChIP experiment (Mock), an unrelated control IgG (against chicken IgY) was used. Experiments were performed in triplicate; bars indicate standard deviation.

PRMT5 is the main type-II PRMT in the nucleus of MEFs that targets histone H4 and is responsible for H4R3me2s. We asked whether allele-specific recruitment of PRMT5 to the chromatin could be responsible for the observed allelic H4R3me2s at ICRs. Real-time PCR amplifications revealed a degree of PRMT5 binding to ICRs that was comparable to that observed at the *CyclinE1* promoter (Figure 5A and B), at which we reported binding previously (10). At two different ICRs (KvDMR1 and *Snrpn*), we sequenced the PRMT5 ChIPed DNA and SNPs showed that PRMT5 was preferentially precipitated on the DNA-methylated allele (Figure 5C). Furthermore, H4R3me2s levels were reduced at all ICRs tested in the *Prmt5* knock-down MEFs, and a marked reduction was observed at IAP elements as well (Figure 5D). However, imprinted gene expression was retained in the *Prmt5* knock-down MEFs (Supplementary Figure S3A), arguing against a role of H4R3me2s in the allelic repression of imprinted genes. We conclude from these data that PRMT5 is recruited to the DNA methylated allele of ICRs on which it methylates H4R3me2s.

Next, we asked whether a PRMT5 partner protein, called COPR5, previously reported to recruit PRMT5 to the *Cyclin E1* promoter (34), could recruit PRMT5 onto the chromatin of ICRs as well. We found that in newly derived *Copr5* knock-out MEF cells (E. Fabbrizio, unpublished results), the overall PRMT5 levels were decreased in the nucleus. In the COPR5-deficient cells, however, the global histone H4R3me2s levels remained unaffected. This indicates that COPPR5 levels do not impact significantly on the bulk of PRMT5-mediated H4R3me2s on the chromatin (Supplementary Figure S3B). However, consistent with our previous data (34), PRMT5 recruitment was decreased at specific promoters, such as the *Cyclin E1* promoter. Interestingly, decreased PRMT5 binding was not observed at the KvDMR1 ICR region (Supplementary Figure S3C), arguing against a role of COPR5 in the recruitment of PRMT5 to this ICR. Possibly another partner protein could be involved in the recruitment of PRMT5 to the DNA-methylated alleles of ICRs.

### PRMT5-mediated H4R3me2s is independent from H3K9me3, H4K20me3 and DNA methylation at imprinted gene loci

**Figure 6.**
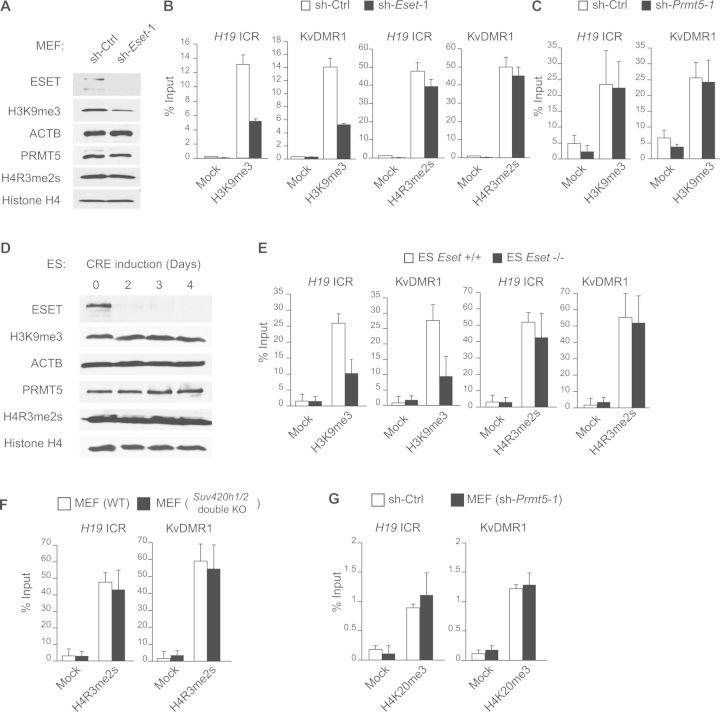
H4R3me2s is regulated independently from H3K9me3 and H4K20me3 at imprinted loci. (**A**) Knockdown of ESET by retroviral shRNA in MEF cells (line sh-*Eset*-1). Levels of ESET, H3K9me3, PRMT5 and H4R3me2s were assessed by western blotting; ACTB and H4 are included as internal controls. As a negative control, MEFs stably expressing a scrambled shRNA were analysed (line sh-Ctrl). (**B**) ChIP-qPCR shows loss of H3K9me3 at the *H19* and KvDMR1 ICRs in the sh-*Eset*-1 MEFs. H4R3me2s levels are unaltered. Experiments were performed in triplicate; bars indicate standard deviation. (**C**) In PRMT5-depleted MEFs (line sh-*Prmt5-*1), H3K9me3 levels are unaltered at *H19* and KvDMR1. (**D**) Conditional knockout of *Eset* in ES cells through a Cre-recombinase and loxP system (17). Levels of ESET, H3K9me3, PRMT5 and H4R3me2s were assessed by western blotting. (**E**) ChIP-qPCR shows loss of H3K9me3 at the *H19* and KvDMR1 ICRs in ESET knockout ES cells. H4R3me2s levels are unaffected. Mock ChIPs were as described for Figure 5G. (**F**) In *Suv4-20h1/h2* double knockout MEFs, H4R3me2s levels assessed by ChIP-qPCR are not affected at the *H19* and KvDMR1 ICRs. (**G**) H4K20me3 levels are unaltered at ICRs in PRMT5-depleted MEFs (line sh-*Prmt5*-1). Experiments were performed in triplicate; bars indicate standard deviation. Mock ChIPs were as described for Figure 5G.

At ICRs, H4R3me2s was consistently detected on the same parental allele as H3K9me3 (Figure 4G). We therefore explored whether H3K9me3 could contribute to the somatic maintenance of H4R3me2s. ESET (also called SetDB1, KMT1E) is a candidate lysine-methyltransferase (KMT) for the allelic H3K9me3 at ICRs. In a recent genome-wide ESET binding assay, this SET domain-containing protein had been reported to bind to several ICRs, including the *H19* ICR (35). Because complete loss of *Eset (Setdb1)* expression induces death in differentiated cells (36), we used an shRNA-based approach to strongly, but not completely, reduce ESET levels (35). One out of several retroviral constructs tested, sh-*Eset-1*, induced a marked depletion of ESET in the MEFs (Figure 6A). As expected, this led to a partial, but significant, reduction in overall H3K9me3 levels, as detected by western blotting. In contrast, the overall levels of H4R3me2s remained unaltered in the knock-down cells. At two ICRs analysed, KvDMR1 and *H19* ICR, there was a marked reduction in H3K9me3, but not in H4R3me2s levels in the sh*-Eset-1* cells compared to control MEFs (sh-Ctrl) (Figure 6B). Next we analysed mouse ES cells with a conditional *Eset* allele, for which we showed previously that ESET controls H3K9me3 at lineage differentiation markers (17). Similarly as in the knock-down MEFs, inducible CRE-mediated loss of ESET in ES cells (Figure 6D) led to reduced levels of H3K9me3 at the KvDMR1 and *H19* ICRs, but again, without a noticeable loss of H4R3me2s (Figure 6E). Combined, these data indicate that ESET controls H3K9me3 at ICRs in MEFs and ES cells, and that its depletion does not affect H4R3me2s at ICRs.

Next, we addressed the opposite question; namely, whether H3K9me3 is affected by the loss of PRMT5. As reported by others (37), we observed by western blotting that there was no overall reduction in H3K9me3 levels in the PRMT5-depleted MEF cells (Figure 1E). Similarly, we did not observe reduced H3K9me3 precipitation at the *H19* and KvDMR1 ICRs (Figure 6C). These data suggest that PRMT5 and H4R3me2s are not essential for the somatic maintenance of H3K9me3 in MEFs.

Chromatin at the repressed alleles of ICRs is also consistently marked by H4 lysine-20 trimethylation (H4K20me3) (38). Previously, we showed that the maintenance of H4K20me3 at the KvDMR1 and *H19* ICRs is controlled by the combined action of SUV4-20H1 and SUV4-20H2 (Suv4-20H1/H2, also called KMT5B/C). These KMTs are recruited to the chromatin on the repressed alleles of ICRs (26). *Suv4-20h1/h2* double-knockout cells (18) are viable and do not show loss of imprinting (26). Absence of SUV4-20H1 and SUV4-20H2 had also no apparent impact on global H4R3me2s levels, and did not alter H4R3me2s at the KvDMR1 and *H19* ICRs either (Figure 6F). This result indicates that SUV4-20H1 and SUV4-20H2 do not interfere with the PRMT5-mediated H4R3me2s at imprinted target loci. Conversely, in the sh-*Prmt5*-1 knock-down cells, there was no apparent reduction in H4K20me3 at ICRs (Figure 6G).

Finally, we determined whether the allelic DNA methylation patterns at ICRs were altered in *Prmt5* knock-down cells. There was no evidence for altered DNA methylation at the *H19* ICR, IG-DMR, Peg3 ICR and *Snrnp* ICRs (Supplementary Figure S4). CpG methylation at IAP elements was not altered either. Thus, the loss of H4R3me2s in the experimental MEFs had not affected the maintenance of CpG methylation at ICRs, nor the H3K9me3 and H4K20me3 chromatin marks associated with this DNA methylation.

Our combined knock-down and knockout studies suggest that PRMT5-mediated H4R3me2s does not depend on ESET-mediated H3K9me3 or SUV4-20H1/H2-mediated H4K20me3 at imprinted target loci. Conversely, ESET-mediated H3K9me3, SUV4-20H1/H2-mediated H4K20me3 and DNA methylation seem not to depend on PRMT5-mediated H4R3me2s. Though the three chromatin-modifying systems act on identical target regions such as ICRs and IAP elements, PRMT5-mediated H4R3me2s seems to be regulated independently from the two repressive lysine methylation pathways.

Although our data provide novel insights into the regulation and epigenomic distribution of H4R3me2s, they leave open the question as to what could be the function of this covalent histone modification in chromatin. However, there seems not to be a general link with gene repression or gene expression. As a first step to address this question, we re-analysed 29 published ChIP-seq data sets together with our ChIP-seq data for H4R3me2s in ES cells and evaluated the genome-wide correlations between them (see Supplementary Table S4 for the list of data used and Figure S6 for the analysis). This confirmed the genome-wide correlation of H4R3me2s with methylated CpG dinucleotides and with hydroxymethylated CpGs (Pearson’s *r* = 0.3, 0.33, respectively; see also Figure 3F). Interestingly, besides a link with H3K4me3 at promoter regions (*r* = 0.29), we also detected a significant correlation with the binding of two proteins of the Mediator complex (Med1 and Med12) (*r* = 0.29 and 0.28), and with the cohesin loading protein Nipbl (*r* = 0.31) and with two cohesin proteins (Smc1 and Smc3) (*r = *0.25 and 0.24). Interestingly, the latter factors are all involved in DNA configuration and looping, such as between enhancer and promoters (39), suggesting that in part H4R3me2s is enriched at regions involved in chromatin contacts.

## DISCUSSION

The main findings of this study is that PRMT5 controls H4R3me2s in embryonic fibroblasts and that H4R3me2s peaks are largely confined to G + C-rich regions, including most of the genome’s promoters/CpG islands. Our biochemical studies identify PRMT5 as the main type-II enzyme for H4R3 and H2AR3 in embryonic fibroblasts. This function of PRMT5 complements the earlier demonstration that PRMT5 methylates H3R8 and H4R3 at specific target loci, including repressed tumour suppressor genes (9,40). The pronounced nuclear presence of PRMT5 in fibroblasts compared to ES cells seemed unrelated to the observed patterns of H4R3me2s enrichment along the genome, which were similar between MEFs and ES cells. However, some statistically defined peaks were identified in ES cells but not in MEFs. This difference between undifferentiated and differentiated cells may come from the expression of PRMT7 in ES cells, which could potentially also regulate H4R3me2s, either directly (41,42) or indirectly via the production of H4R3me1 (43). This possibility would need to be addressed in future studies.

Unexpectedly, our data did not reveal a general correlation between gene expression and H4R3me2s enrichment. Indeed, the vast majority of annotated mouse promoters are marked by H4R3me2s in both ES cells and MEFs, including actively transcribed and repressed genes. This novel insight changes our view on the possible role(s) of H4R3me2s, which in earlier studies on established human cell lines was found to be associated with gene repression (6,8,11). One characteristic of chromatin surrounding active promoters is that many comprise the non-canonical H3 variant H3.3 (44), whereas the H2A variant H2A.Z is present at mitotically silenced genes (45). In future research, it should be relevant to explore whether a non-canonical nucleosomal organization, possibly in combination with specific histone modifications, interferes with the acquisition and/or maintenance of H4R3me2s. One modification that could interfere with H4R3me2s acquisition would be asymmetrical dimethylation of H4R3 (H4R3me2a). Recent *in vitro* studies on the transcriptional co-activator TDRD3—a Tudor-domain protein that binds to H4R3me2a—show that this protein binds to the TSS regions of certain genes in human breast cancer cells (46); however, no significant enrichment was detected on the non-methylated alleles of human ICRs. For technical reasons, so far no studies have reported the genome-wide patterns of H4R3me2a, but such data should be interesting in comparison to the H4R3me2s profiles obtained in the current study. Recently, an inverse correlation between H4R3me2s and MLL4-mediated H3K4me3 was reported at the *HOXA* and *HOXB* promoters in human cells (47). Although we found H4R3me2s enrichment at the *Hoxa* and *Hoxb* promoters in mouse ES cells as well, it is unlikely that H4R3me2s and H3K4me3s are mutually exclusive since we detected co-enrichment of the two marks at all active promoters in ES cells. Another recent study reported H2A/H4R3me2s association with satellite repeats, based on a rather limited, filtered ChIP-seq data set (only 1845 nucleosomes were analysed in total) (48). In our ChIP-seq experiments, we did not observe significant H4R3me2s enrichment at satellite-II or III sequences, which are G + C-poor repeats (Supplementary Table S3 and Figure S5). In contrast, there was a general H4R3me2s enrichment at IAP elements, which are long tandem repeats of higher G + C content, and this finding confirms our earlier findings in embryos and primordial germ cells (49).

Although H4R3me2s did not generally correlate with transcriptional activity or DNA methylation levels, it was consistently linked to the presence of CpG methylation at specific loci. These included IAP retro-transposons (Supplementary Figure S4) and ICRs at imprinted gene domains. We used the latter, allelic model system to carefully explore the link between H4R3me2s and repressive histone lysine methylation. PRMT5-mediated H4R3me2s was detected on the DNA-methylated alleles of ICRs only and was found to be independent from the repressive histone lysine methylation controlled by the SET-domain proteins ESET and SUV4-20H1/H2. What dictates the allelic PRMT5-mediated H4R3me2s at ICRs remains unclear. It should be interesting to explore whether this process could be linked to other histone modifications or to allele-specific higher order chromatin features at these unusual regulatory elements.

PRMT5 and H4R3me2s were shown previously to be associated with methylated CpG dinucleotides in cultured human cells, through interaction of PRMT5 with MBD2 (Methyl CpG Binding Domain 2) (MCF7) (50). Another study reported that H4R3me2s serves as a direct binding target for DNA methyltransferase DNMT3A (8), suggesting that PRMT5-mediated methylation of H4-arginine3 could be necessary for the establishment of *de novo* DNA methylation. In agreement with this hypothesis, we and others reported that ICRs acquire high levels of H4R3me2s in developing male germ cells prior to and during the acquisition of DNA methylation (41,49). In another study, by Otani *et al.* (51), however, no evidence was obtained for interaction of the ADD (ATRX-DNMT3A-DNMT3L) domain of DNMT3A with an H4R3me2s peptide. These different observations prompted us to ask whether H4R3me2s could be necessary for DNA methylation at ICRs in somatic cells. However, no loss of CpG methylation was observed at ICRs, or at IAPs, in the PRMT5 knock-down cells suggesting that DNA methylation maintenance might be independent from H4R3me2s. Altogether, our data indicate that H4R3me2s is a PRMT5-controlled histone mark which accumulates at G + C-rich sequence elements and at repressed alleles of ICRs in somatic cells. This process is generally independent from the transcriptional activity or the occurrence of repressive chromatin marks.

Finally, our preliminary bioinformatic comparison with published ChIP-seq data reveals an intriguing link between H4R3me2s-enrichment and recruitment of core components of the Mediator complex and cohesin proteins at gene regions. As concerns the Mediator complex, one way this has been reported to interact with PRMT5 is through its associated cyclin-dependent kinase subunits (52). Although this needs to be explored in future studies, the observed link with cohesins and mediator complexes suggests that PRMT5-mediated H4R3me2s may directly or indirectly facilitate higher order chromatin contacts.

## SUPPLEMENTARY DATA

Supplementary Data are available at NAR Online including [53–65].

## FUNDING

Agence Nationale de la Recherche (ANR), the Institut National Contre le Cancer (INCa), La Ligue Contre le Cancer and the Agency for International Cancer Research (AICR, UK); We acknowledge post-doctoral fellowships from the Japanese Society for the Promotion of Science (JSPS) (to R.H.), the ‘Ligue Contre le Cancer’ (France) (to M.G.) and the University of Montpellier-I (to M.G.); affiliated to the European network EpiGeneSys and the LABEX EpiGenMed (to R.F.). Funding for open access charge: INCA (French National Cancer Institute).

*Conflict of interest statement*. None declared.

## Supplementary Material

Supplementary Data
