# Supplementary material for: PRMT5-mediated histone H4 arginine-3 symmetrical dimethylation marks chromatin at G + C-rich regions of the mouse genome
Source: Nucleic Acids Res. 2013 Oct 3;42(1):235–48. doi: 10.1093/nar/gkt884 (PMC3874197; doi:10.1093/nar/gkt884)
Supplement: Supplementary Data [file supp_42_1_235__index.html]

PRMT5-mediated histone H4 arginine-3 symmetrical dimethylation marks chromatin at G + C-rich regions of the mouse genome — PRMT5-mediated histone H4 arginine-3 symmetrical dimethylation marks chromatin at G + C-rich regions of the mouse genome — Supplementary Data 

# PRMT5-mediated histone H4 arginine-3 symmetrical dimethylation marks chromatin at G + C-rich regions of the mouse genome

## Supplementary Data

files

**Files in this Data Supplement:**

- Supplementary Data - pdf file
