# Supplementary material for: PRMT5-mediated histone H4 arginine-3 symmetrical dimethylation marks chromatin at G + C-rich regions of the mouse genome
Source: Nucleic Acids Res. 2013 Oct 3;42(1):235–48. doi: 10.1093/nar/gkt884 (PMC3874197; doi:10.1093/nar/gkt884)
Supplement: Supplementary Data [file supp_gkt884_nar-01691-x-2013-File008.pdf]

Figure S1

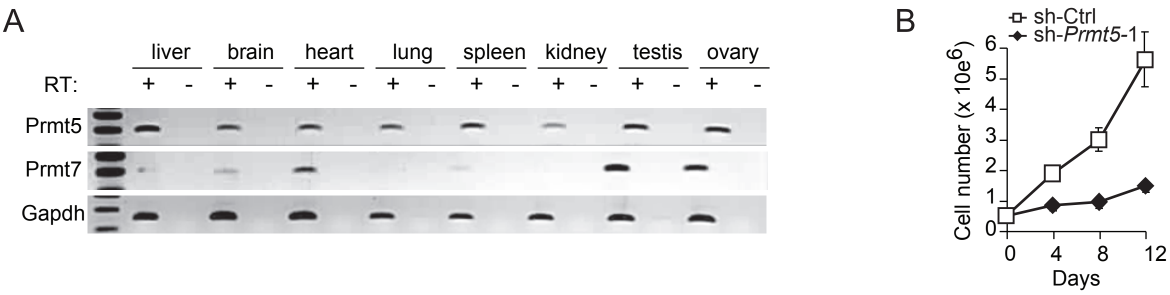

Supplementary Figure S1. (**A**) Expression of *Prmt5* and *Prmt7* in adult mouse tissues. Total RNAs were extracted from mouse adult tissues. After reverse transcription using random oligonucleotides, obtained cDNAs were amplified with primers specific for *Prmt5* and *Prmt7*. In this semi-quantitative approach, *Prmt5* expression is readily detected in all tissues. *Prmt7* expression is detected in specific tissues only, most strongly in testis and ovary. *Gapdh* was used as an internal control. + and - indicate reverse transcriptase positive and negative, respectively. Left lanes include a kb size marker to verify the specificity of PCR amplification. (**B**) Cellular proliferation of *Prmt5* knockdown MEFs. Following retroviral infection, and after 48 hours of selection with puromycin, cells were re-seeded onto new 10cm culture dishes. (see Methods). Cells were counted at days 0, 4, 8 and 12, respectively.

Figure S2

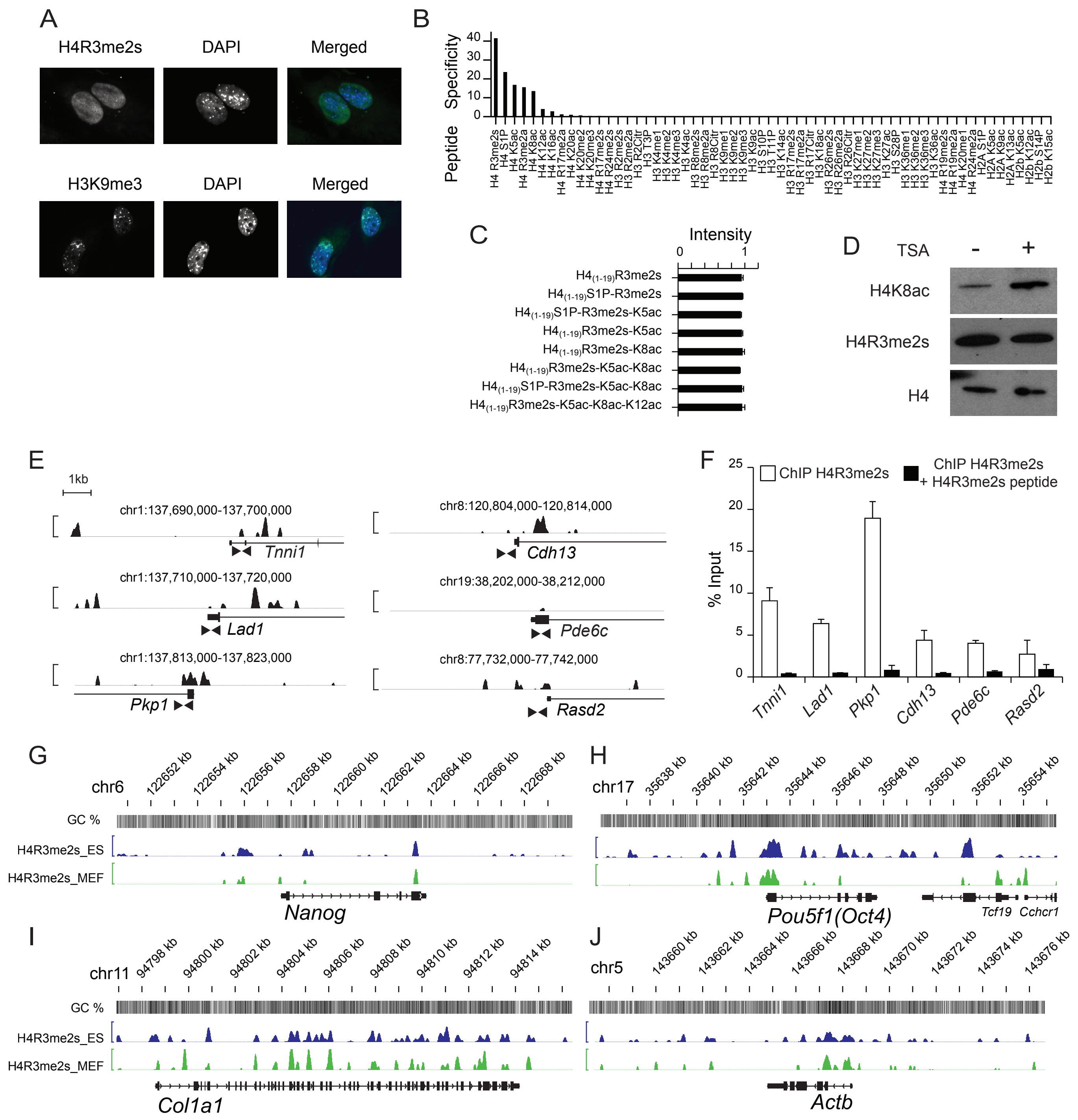

Supplementary Figure S2. (**A**) Immuno-fluorescent staining of H4R3me2s in MEFs. The H4R3me2s signal (upper left) is not detected as specific nuclear foci, but rather, is broadly present across the nucleus in MEF cells. By contrast, H3K9me3 (lower left) accumulates in DAPI-positive nuclear foci. In the merged images, blue and green indicate DAPI and the specific antibody staining, respectively. (**B**) Analysis of the specificity of the H4R3me2s antiserum-2 against different modified peptides spotted on the MODified™ Histone Peptide Array (Active motive ref. 13001). The specificity is defined as the ratio of the average of the chemiluminescent intensities of all spots containing the mark versus the average intensity of all spots not containing the mark. (**C**) Effects of neighbouring modifications on H4R3me2s antiserum-2 binding specificity. (**D**) Detection of H4R3me2s in Trichostatin-A treated 3T3 cells. Western blotting of 3T3 cells treated for 6h with the HDAC-inhibitor TSA (300 nM), precisely as reported before (52). After the treatment with TSA, proteins were extracted under acidic conditions. In TSA-treated cells, there is acquisition of global histone acetylation including on all lysines of the N-terminal tail of histones H4 (K5, K8, K12, K16) (51), as shown for H4 lysine-8 acetylation (H4K8ac). Western detection of H4R3me2s (anti-serum 2) is unaffected in the TSA-treated cells. (**E**) H4R3me2s ChIP-seq profile in MEF cells at selected promoters. (**F**) Chromatin immunoprecipitation levels (% Input) were assesed by real-time PCR at H4R3me2s-marked genomic regions identified by ChIP-seq using an antibody against H4R3me2s (white, "anti-serum 2", Supplementary Table S1). Precipitation was inhibited in the presence (black) of an H4R3me2s-blocking peptide (2 µg/ml; Abcam ab14791). (**G, H, I, J**) ChIP-seq profiles at selected genes. *Nanog* (**G**) and *Pou5f1*(*Oct4*) (**H**) are expressed in ES cells but not in MEF cells, however the H4R3me2s precipitation is similar in the two cells lines. *Col1A1* (**I**) is a gene expressed in MEFs but not in ES cells, *Actb* (**J**) is expressed in both cell types. Heatmaps representing the G+C percentage in 5 bp windows are shown above the maps.

Figure S3

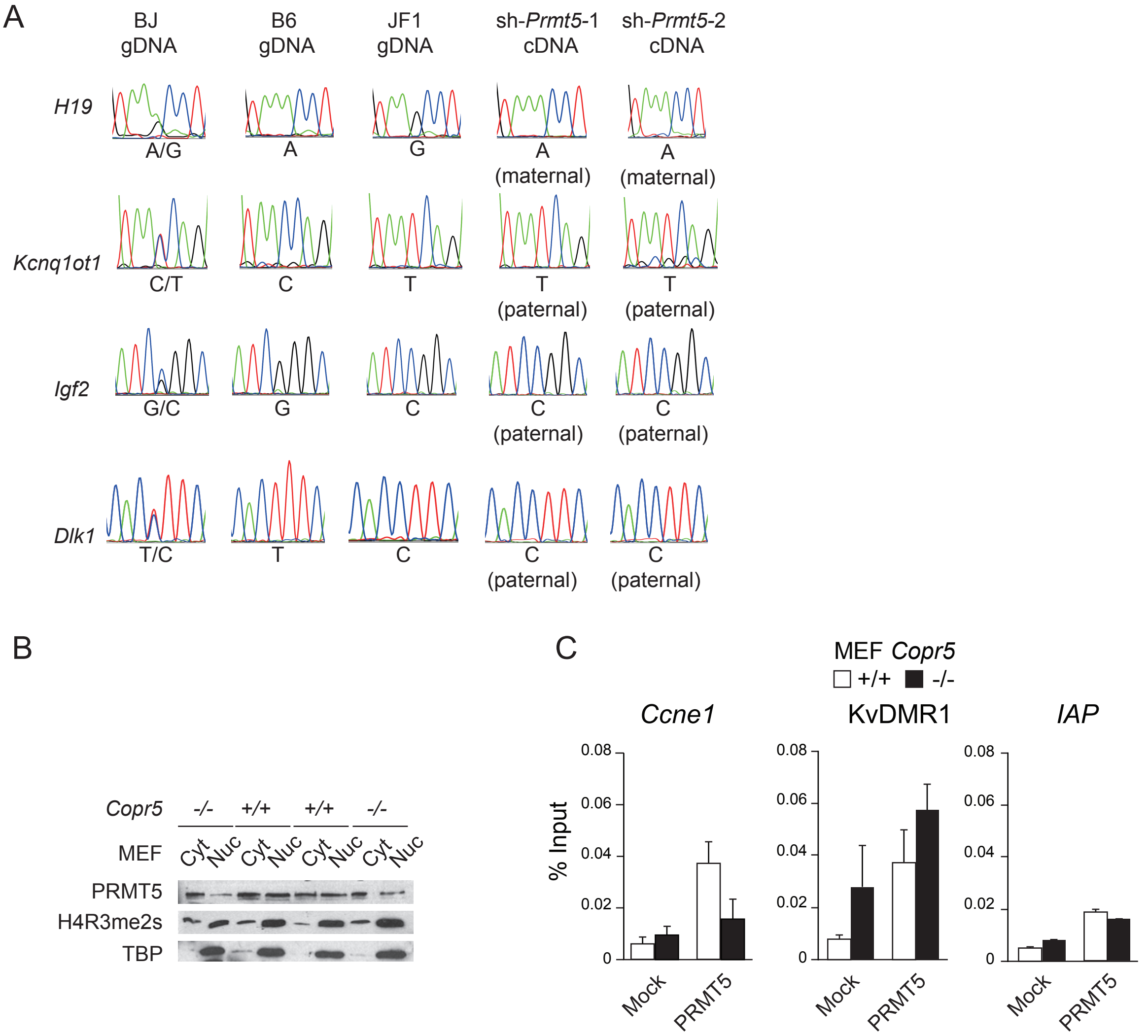

Supplementary Figure S3. (**A**) *Prmt5* knock-down does not affect imprinted gene expression. Sequences of single nucleotide polymorphisms in the indicated transcripts subjected to imprinting (*H19, Kcnq1ot1, Igf2* and *Dlk1*). Sequences of the same regions in the genomic DNA are reported as positive controls (B6: maternal genome; JF1: paternal genome). The sequencing of the cDNAs from *Prmt5* knock-down MEF cells (sh-*Prmt5*-1 and sh-*Prmt5*-2) revealed no changes in the allelic expression of *H19* (maternal transcript),*Kcnq1ot1* (paternal transcript), *Igf2* (paternal transcript) or *Dlk1* (paternal transcript). (**B**) *Copr5* knock-out does not affect PRMT5 detection at ICRs. Western blotting of cytoplasmic (Cyt) and nuclear (Nuc) extracts from MEF cells derived from *Copr5 -/-* or WT (+/+) embryos. PRMT5 is decreased in the nucleus in absence of COPR5 while H4R3me2s nuclear levels are unaffected. TBP is used as a positive control for the nuclear fraction. (**C**) Chromatin immuno-precipitation in *Copr5 -/-* cells shows a decreased PRMT5 recruitment at the *Cyclin E1* (*Ccne1*) promoter, but not at the KvDMR1 ICR and IAP elements.

Figure S4

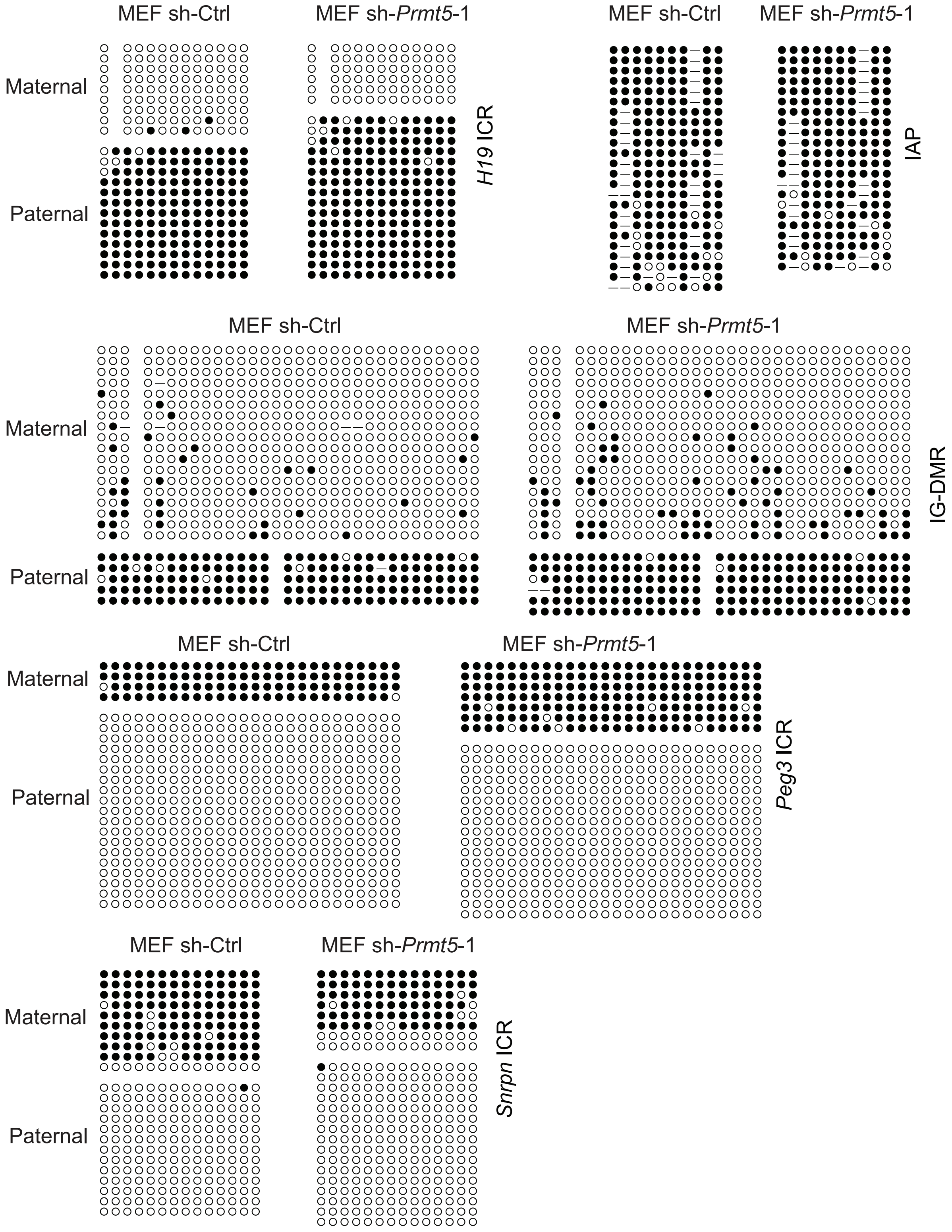

Supplementary Figure S4. Unaltered DNA methylation at ICRs in *Prmt5* knockdown cells. DNA methylation was analysed by bisulfite sequencing. Briefly, genomic DNAs from *Prmt5*-knockdown (sh-*Prmt5*-1) and sh-Ctrl MEFs were mixed directly into a 10M Sodium bisulfite solution (to obtain a 9 M final solution, as described by Shiraishi and Hayatsu (53), denatured at 98°C for 1 min, and incubated for 1 hour at 70 °C. Desulfphonation and purification of bisulfite-treated DNA were performed using a 'Bisulfite DNA purification kit' (Zymo Research). Primer sequences are provided in Supplementary Table 2. Following PCR amplification, maternal and paternal parental alleles were distinguished using SNPs. Closed and open circles indicate methylated and unmethylated CpG dinucleotides, respectively. DNA methylation was unaltered in the *Prmt5* knockdown MEFs at all ICRs analysed and of IAP elements.

Figure S5

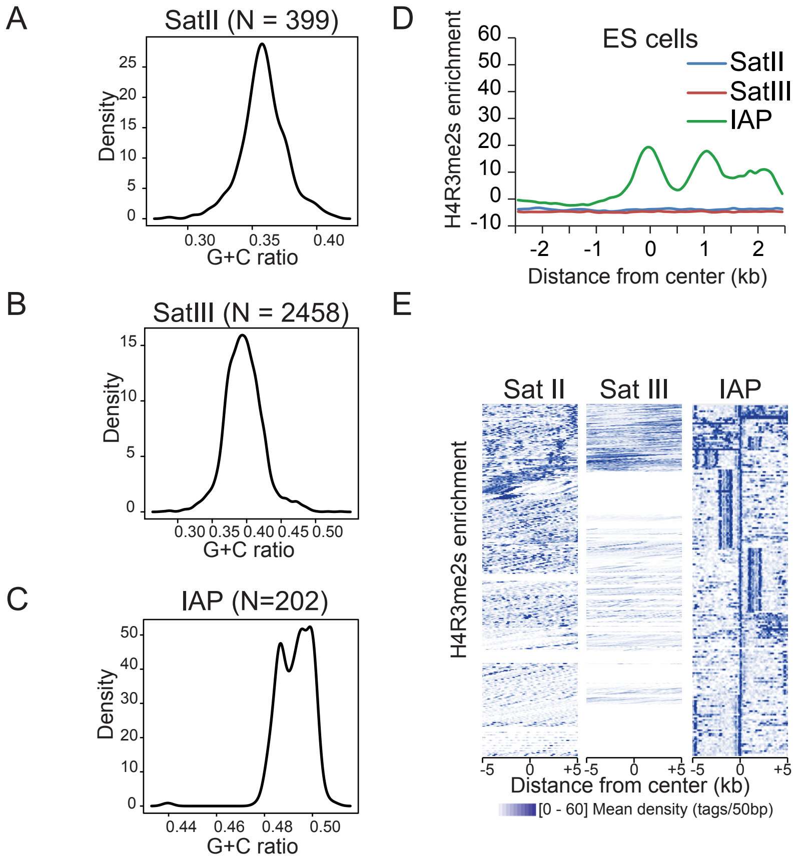

Supplementary Figure S5. H4R3me2s is enriched at G+C-rich repeat elements. (**A,B,C**) Distribution of averaged G+C ratios for type II and III satellite repeats (Sat II, Sat III) and IAP elements, showing that the majority of SatII and III have a G+C ratio at around 0.35-0.40, while IAPs are richer in G+C with a ratio peaking at around 0.5. (**D**) Average H4R3me2s enrichments in 5kb windows around the center of satellite II (Sat II in blue), satellite III (Sat III in red) and IAP (in green) elements in ES cells. These aligned ChIP-seq enrichments are depicted according to the 5'-3' orientation of each repeat element. (**E**) Heatmap profiles of H4R3me2s enrichments in ES cells. The clustered genomic positions do not take into account the orientation of repeat elements.

Figure S6

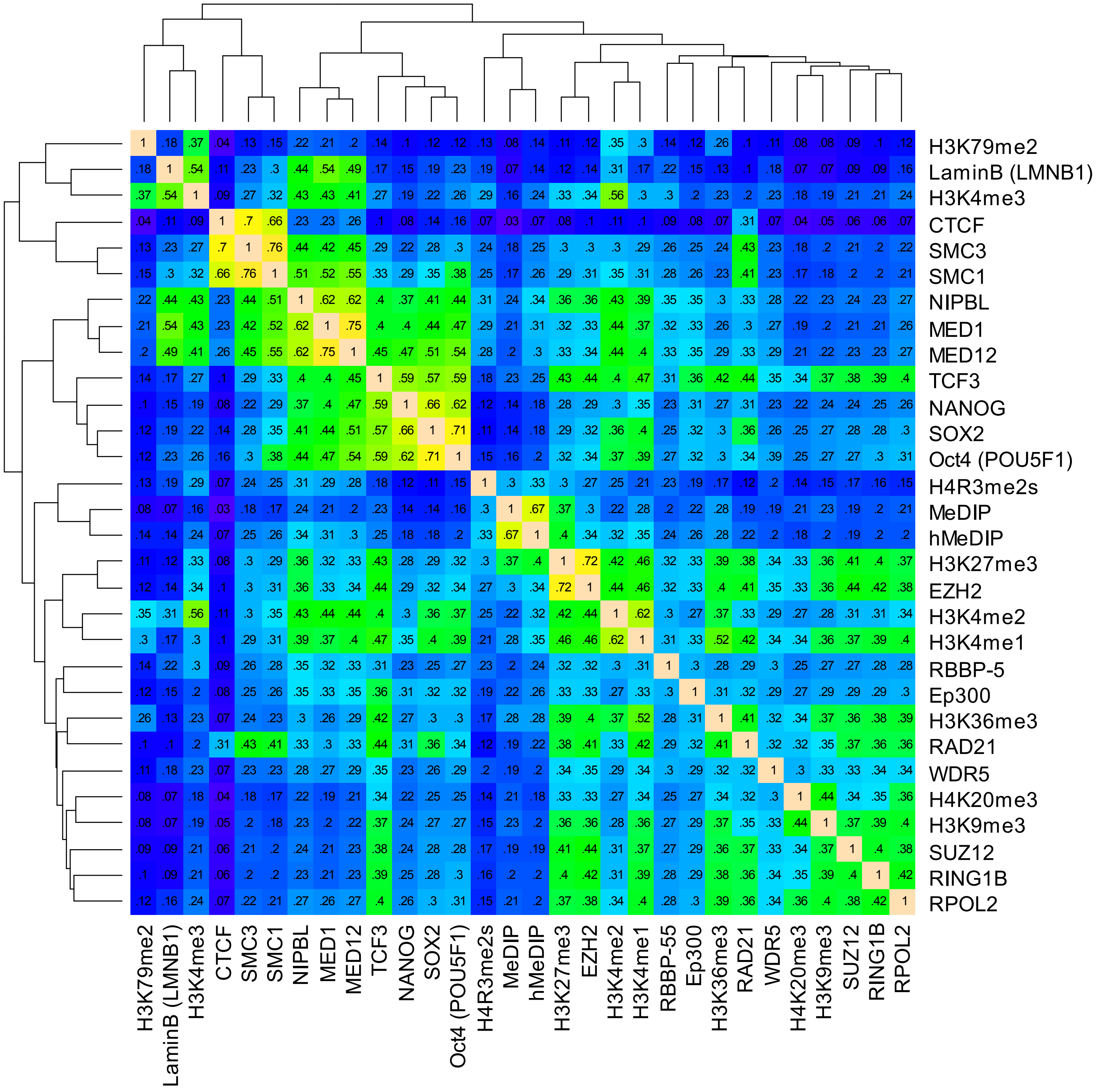

Supplemental Figure S6. Genome-wide correlations of H4R3me2s enrichments with 29 published ChIP-seq data sets in mouse ES cells. The references and accession numbers of the ChIP-seq data used are indicated in the Supplementary Table S4. The numbers indicate Pearson's product-moment correlation coefficients (*r*) of each ChIP-seq comparisons. Briefly, the raw sequencing files were downloaded and replicates were combined into a single file (fastq). After alignment on the mouse reference genome (mm9) with Bowtie 0.12.9, the genomic coordinates were used to compute the genome-wide occupancy density with MACS 1.4.2. These density files (wig file, step=10bp) were then normalized by the total amount of aligned reads (RPM: reads per million) and the Pearson's product-moment correlation coefficients (*r*) for each pair were computed with the R programming language with the "rcorr" library. The matrix of Pearson's coefficients was then represented as a heatmap together with its dendrograms representing the hierarchical clustering of rows (side) and columns (top). This global analysis indicates that H4R3me2s enrichments correlate mostly with methylated and hydroxymethylated CpG (MeDIP, hMeDIP) enrichments, H3K4me3 enrichments, and with Mediator and cohesin proteins enrichments (MED1, MED12, NIPBL, SMC1 and SMC3).

Supplementary Table S1

Antiserum used in this study

**Antigen**	**Producer**	**Catalog number**	**Application**
Histone H3K4me2	millipore	07-030	ChIP
Histone H3K4me3	abcam	ab8580	Western blotting
Histone H3K9me3	millipore	07-442	ChIP, Western blotting
Histone H4	millipore	07-108	Western blotting
Histone H4R3me2s (anti-serum 1)	abcam	ab5823 (batch n° 520317)	Western blotting, Immunostaining
Histone H4R3me2s (anti-serum 2)	abcam	ab5823 (batch n° 885940)	ChIP, Western blotting
Histone H4K8ac	millipore	07-328	Western blotting
Histone H4K20me3	millipore	07-463	ChIP, Western blotting
PRMT5	millipore	07-405	ChIP, Western blotting, Immunostaining
PRMT7	santa cruz	sc-48660	Western blotting
ESET/SetDB1	millipore	07-378	Western blotting, Immunostaining
ESET/SetDB1	santa cruz	sc-66884	ChIP
NFkB/p65(RELA)	santa cruz	sc-372	Immunostaining
β Actin (C4)	santa cruz	sc-47778	Western blotting
α Tubulin	sigma	T9026	Western blotting
Chicken IgY	Sigma	C-2288	ChIP

Supplementary Table S2

Primers for qPCR and allelic analysis after ChIP

Region	Application	Primer sequence	Chromosome	start-end	length	Reference
H19 ICR	SSCP, qPCR	GATCAGGCATTTGTGCACTTAC<br>TAGGTTACCTGGGACATTGC	chr7	149765926<br>149766128	203bp	(49)
H19 ICR	Sequencing	ATGCCAGAAAGCACAAAAGC<br>CTCGGCAACTTCGGTCTTAC	chr7	149766457<br>149766997	541bp	This study
H19 downstream (region a)	Sequencing	ATTCATAGAGACAGCTCAAGGTCC<br>TAATGCTAGTCCGGCTTCCAGATC	chr7	149751500<br>149752081	582bp	This study
H19 upstream (region b)	Sequencing	GGAATTCATCAAGCAGTTTGAGCC<br>TCCCTGTCTGGAATGCACATGTGG	chr7	149783678<br>149784197	520bp	This study
IG-DMR	qPCR	GAAGACAAAGAGCAAGCCTGT<br>TAGACAACGGTGAGCCAGGAT	chr12	110767554<br>110767750	197bp	(38)
IG-DMR	Sequencing	CGCAGGTCCTAACACGGCAC<br>GAGCGTTGGAGCCTTGAGCC	chr12	110768064<br>110768490	427bp	This study
KvDMR1	SSCP, qPCR, Sequencing	CGGATCACTTGAGCACTAC<br>GCCAAGTGGATCGCGCCAAG	chr7	150481890<br>150482128	239bp	(49)
Rasgrf1 ICR	qPCR, Sequencing	AGGCACTACCTTGCCTGGTTTA<br>GCAAAAAGGAGGCGTTTATCTG	chr9	89774003<br>89774194	192bp	(38)
Snrpn ICR	qPCR, Sequencing	AGGTTGTGACTGGGATCCTG<br>TGCAGCGGCAACAGAACTTCT	chr7	67149483<br>67149715	233bp	(49)
IAP	qPCR	TATGCCGAGGGTGGTTCTCTA<br>TGCGGCAAAACTTTATTGCTT	multiple	multiple	~200bp	(49)
Tnni1	qPCR	TGTTGGGTCTTGAGGGAAAC<br>GAGAGCATCCTCCACTCTGC	chr1	137696239<br>137696314	76bp	This study
Lad1	qPCR	CCCCACTTCCTGTTCAGTGT<br>GGAGGGGCCATTTTTGTAAT	chr1	137714336<br>137714426	91bp	This study
Pkp1	qPCR	GAGCACTCACACCTCCTTCC<br>CCCTGGGATCCCTTGATATT	chr1	137814665<br>137814781	117bp	This study
Cdh13	qPCR	GTGTGGTTGGCCTTGGTACT<br>ATGGCTCCCCCTCCTACTAA	chr8	121406234<br>121406338	105bp	This study
Pde6c	qPCR	TGCCTTTGGGTAACAGGTTC<br>TGACAGGACGGTACCCTTTC	chr19	38236748<br>38236812	65bp	This study
Rasd2	qPCR	CTCGCTGAGCTAGGAGGCTA<br>GTCCCCAGGGTAGGTCTCTC	chr8	77742762<br>77742866	105bp	This study
Col1a	qPCR	CCGGGCTCTGATTTGCTG<br>GACCAGGAGTGCGGACCT	chr2	27741844<br>7741985	142bp	This study
Actb	qPCR	CGCTGTGGCGTCCTATAAAA<br>AAGGAGCTGCAAAGAAGCTG	chr5	143668357<br>143668446	90bp	This study
Nav1	qPCR	CGAATGCTCAGACTTGGACA<br>TGAGCCTGAGTGGAGTGATG	chr1	137481454<br>137481568	115bp	This study
Csrp1	qPCR	CGGTCCAGGTGTTGTCTTTT<br>TACCCTCTCAGCCTGCAACT	chr1	137627377<br>137627460	84bp	This study
Phlda3	qPCR	AACCAGGGGCTTTGATTTCT<br>TTGGTCCCAACAACCTTCTC	chr1	137663527<br>137663588	62bp	This study
Tnnt2	qPCR	AGTGGGTGTTTCCAGGACAG<br>CAAGCACTCTCCACTCCACA	chr1	137733187<br>137733242	56bp	This study

Primers for RT-PCR

Gene	Application	Primer sequence	Chromosome	start	length	Reference
Prmt5	RT-PCR	TGAACACAGTGCTTCATGGCTTCG<br>GAATTGCTGCATCGCCAGAAACGC	chr14<br>chr14	55128180<br>55126723	193bp	This study
Prmt7	RT-PCR	ACTGTGGGAATACCCCTGCAGAAG<br>TTTATGAGGCCAGTGCTGATCGTG	chr8<br>chr8	108774252<br>108775177	198bp	This study
Eset/SetDB1	RT-PCR	GCAATGGAGAAGAAGCAAGG<br>ATAGGCTGTAGGGGCTCCAT	chr3<br>chr3	95144141<br>95145304	143bp	This study
Gapdh	RT-PCR	TGTGTCCGTCGTGGATCTGA<br>TTGCTGTTGAAGTCGCAGGA	chr6<br>chr6	125112535<br>125112302	150bp	This study

Primers for bisulfite sequencing

Region	Application	Primer sequence	Reference
H19 ICR	Bisulfite sequencing	TGTTATAGTTTTTAGGTTATTTGGGATATTG<br>ACACACATTTCTTAAATAACTCCTTCAATC	(55)
IG-DMR	Bisulfite sequencing	GTGTTAAGGTATATTATGTTAGTGTTAGG<br>TACAACCCTTCCCTCACTCCAAAAATT	(56)
Rasgrf1 ICR	Bisulfite sequencing	GAGAGTATGTAAAGTTAGAGTTGTGTTG<br>ATAATACAACAACAACAATAACAATC	(56)
Snrpn ICR	Bisulfite sequencing	AATTTGTGTGATGTTTGTAATTATTTGG<br>AATAAACCCAAATCTAAAATATTTTAATC	(55)
Peg3 ICR	Bisulfite sequencing	ATGGGGTTTTGGATTGGTTAGAGAGGAAGT<br>ATCTACAACCTTATCAATTACCCTTAAAAA	(55)
IAP	Bisulfite sequencing	TTGATAGTTGTGTTTTAAGTGGTAAATAAA<br>AAAACACCACAAACCAAAATCTTCTAC	(55)

Supplementary Table S3

Details of the sequences alignments on the mouse reference genome mm9.

	Input ES		H4R3me2s ChIP ES		Input MEF		H4R3me2s ChIP MEF	
Total reads	80,285,881	100%	85,325,283	100%	32,305,312	100%	17,253,732	100%
reads with at least one reported alignment	56,891,020	70.86%	34,900,743	40.90%	23,117,861	71.56%	9,886,703	57.30%
reads that failed to align	5,833,393	7.27%	10,969,138	12.86%	2,011,841	6.23%	1,494,439	8.66%
reads suppressed due to multiple alignments (>1)	17,561,468	21.87%	39,455,402	46.24%	7,175,610	22.21%	5,872,590	34.04%
suppressed reads mapping in SINEs	1,126,037	(6.41%)	2,091,760	(5.30%)	467,705	(6.52%)	329,801	(5.62%)
suppressed reads mapping in LINEs	2,185,309	(12.44%)	5,702,363	(14.45%)	878,247	(12.24%)	817,408	(4.74%)
suppressed reads mapping in LTRs	806,219	(4.59%)	1,571,072	(3.98%)	335,082	(4.67%)	239,837	(1.39%)
suppressed reads mapping in Satellites	23,335	(0.13%)	40,133	(0.10%)	9,915	(0.14%)	6,720	(0.04%)

* (percents) indicate percentages relative to suppressed reads

Supplementary table S4

Published ChIP-seq experiments used in the supplementary Figure S6. SRA: Sequence Read Archive (http://trace.ncbi.nlm.nih.gov/Traces/sra/); EBI: European Nucleotide Archive (http://www.ebi.ac.uk/ena/).

Experiment	SRA	EBI	reference
H3H79me2	SRR015144<br>SRR015145		(57)
LaminB (LMNB1)	SRR172855<br>SRR172856		(58)
H3K4me3	SRR006889<br>SRR006828<br>SRR006829<br>SRR006830<br>SRR006831<br>SRR006832		(59)
CTCF	SRR172853<br>SRR172854		(60)
SMC3	SRR058984<br>SRR058983		(63)
SMC1	SRR058981<br>SRR058982		(60)
NIPBL	SRR058989<br>SRR058990		(60)
MED1	SRR058987<br>SRR058988		(60)
MED12	SRR058985<br>SRR058986		(60)
TCF3	SRR015155<br>SRR015156		(60)
NANOG	SRR015149<br>SRR015150		(57)
SOX2	SRR050356<br>SRR050357		(57)
Oct4 (POU5F1)	SRR015151		(57)
MeDIP		ERR031632_1<br>ERR031632_2<br>ERR031634_1<br>ERR031634_2	(24)
hMeDIP		ERR031633_1<br>ERR031633_2<br>ERR031635_1<br>ERR031635_2	(24)
H3K27me3	SRR006787		(59)
EZH2	SRR015128<br>SRR015129		(61)
H3K4me2	SRR002253		(62)
H3K4me1	SRR002255		(62)
RBBP-5	SRR060172		(63)
Ep300	SRR172850<br>SRR172851<br>SRR172852		(58)
H3K36me3	SRR007433<br>SRR007434		(59)

RAD21	SRR065242		(64)
WDR5	SRR060173		(65)
H4K20me3	SRR007437<br>SRR007438		(59)
H3K9me3	SRR007435<br>SRR007436		(59)
SUZ12	SRR015132<br>SRR015133<br>SRR015134		(61)
RING1B	SRR015130<br>SRR015131		(61)
RPOL2	SRR006792		(59)
